# Quantitative Dynamic Contrast-Enhanced Magnetic Resonance Parameters Could Predict International Society of Urological Pathology Risk Groups of Prostate Cancers on Radical Prostatectomy

**DOI:** 10.3390/life13091944

**Published:** 2023-09-21

**Authors:** Chun-Bi Chang, Yu-Chun Lin, Yon-Cheong Wong, Shin-Nan Lin, Chien-Yuan Lin, Yu-Han Lin, Ting-Wen Sheng, Lan-Yan Yang, Li-Jen Wang

**Affiliations:** 1 Department of Medical Imaging and Intervention, Linkou Chang Gung Memorial Hospital, College of Medicine, Chang Gung University, Gueishan, Taoyuan 33305, Taiwan; cooler@cgmh.org.tw (C.-B.C.);; 2 Department of Medical Imaging and Radiological Sciences, Chang Gung University, Taoyuan 33302, Taiwan; 3 Department of Medical Imaging and Intervention, New Taipei Municipal TuCheng Hospital, Chang Gung Memorial Hospital and Chang Gung University, Taoyuan 33305, Taiwan; 4 GE Healthcare, Taipei 11012, Taiwan; 5 Biostatistics Unit of Clinical Trial Center, Chang Gung Memorial Hospital, Gueishan, Taoyuan 33305, Taiwan

**Keywords:** dynamic contrast enhanced, DCE-MRI parameters, ISUP grade, prostate cancer, surgical margin

## Abstract

Background: The International Society of Urological Pathology (ISUP) grade and positive surgical margins (PSMs) after radical prostatectomy (RP) may reflect the prognosis of prostate cancer (PCa) patients. This study aimed to investigate whether DCE-MRI parameters (i.e., *K^trans^*, *k_ep_*, and IAUC) could predict ISUP grade and PSMs after RP. Method: Forty-five PCa patients underwent preoperative DCE-MRI. The clinical characteristics and DCE-MRI parameters of the 45 patients were compared between the low- and high-risk (i.e., ISUP grades III-V) groups and between patients with or without PSMs after RP. Multivariate logistic regression analysis was used to identify the significant predictors of placement in the high-risk group and PSMs. Results: The DCE parameter *K^trans-max^* was significantly higher in the high-risk group than in the low-risk group (*p* = 0.028) and was also a significant predictor of placement in the high-risk group (odds ratio [OR] = 1.032, 95% confidence interval [CI] = 1.005–1.060, *p* = 0.021). Patients with PSMs had significantly higher prostate-specific antigen (PSA) titers, positive biopsy core percentages, *K^trans-max^*, *k_ep-median_*, and *k_ep-max_* than others (all *p* < 0.05). Of these, positive biopsy core percentage (OR = 1.035, 95% CI = 1.003–1.068, *p* = 0.032) and *k_ep-ma_*_x_ (OR = 1.078, 95% CI = 1.012–1.148, *p* = 0.020) were significant predictors of PSMs. Conclusion: Preoperative DCE-MRI parameters, specifically *K^trans-max^* and *k_ep-max_,* could potentially serve as preoperative imaging biomarkers for postoperative PCa prognosis based on their predictability of PCa risk group and PSM on RP, respectively.

## 1. Introduction

Dynamic contrast-enhanced magnetic resonance imaging (DCE-MRI) can provide a valuable and noninvasive method for assessing the complex vascular microenvironment associated with prostate cancers (PCas). The features of PCa growth depend on its need to establish a novel and elaborate vascular network, providing an adequate supply of critical nutrients necessary for tumor sustenance and progression. These newly formed tumor vessels are often primitive in morphology and function [1], which may affect vascularization and capillary permeability in PCa. As a result, PCas usually have greater blood flow than the physiological blood flow observed in normal glandular structures. Prior studies have used DCE-MRI to analyze the higher perfusion of PCa and differentiate it from healthy prostate tissue [2,3,4,5]. Since angiogenesis plays a pivotal role in the clinical course of PCa (i.e., growth, metastasis, and prognosis), the extent of angiogenesis may be a prognostic marker that reflects the aggressiveness of the tumor [6]. The hemodynamic status of angiogenesis can be noninvasively evaluated by DCE-MRI [6]. Therefore, DCE-MRI has emerged as a promising noninvasive technique that holds potential for assessing the complicated angiogenesis within PCa and could serve as a valuable tool for predicting the risk stratification of this malignancy.

Previous studies have shown that DCE-MRI can be used to assess the aggressiveness of PCa [7,8,9,10]. The parameters derived from DCE-MRI may correlate with the Gleason score (GS) and can potentially stratify the risk of PCa. For example, a previous study indicated that the apparent diffusion coefficient (ADC), specifically at a threshold of 0.747 × 10^−3^ mm^2^/s, could effectively distinguish between GS 6 and ≥7 in terms of PCa aggressiveness, achieving 84% diagnostic accuracy with an area under the receiver operating characteristic curve of 0.81 [11]. In the past, the GS was stratified into low-risk (GS 6), intermediate-risk (GS 7), and high-risk (GS 8–10) groups. However, a notable limitation of this tripartite risk stratification is its inability to accurately reflect the substantial heterogeneity demonstrated within the intermediate-risk group (GS 7). In the intermediate-risk group, a significant difference in recurrence has been shown between patients with a GS of 3 + 4 and a GS of 4 + 3; thus, the International Society of Urological Pathology (ISUP) introduced a novel grading system to replace the former three-category risk groups [12,13]. In the novel ISUP grading system, GSs of 6, 3 + 4, 4 + 3, 8, and 9–10 are correspondingly designated grades I through V. With respect to the baseline (ISUP grade I), the hazard ratios for biochemical recurrence progressively advance from 1.9 for grade II, escalating to 5.1 for grade III, 8.0 for grade IV, and finally 11.7 for grade V [12]. Although DCE-MRI may be capable of evaluating the risk of PCa based on GS, the exact correlation between DCE-MRI and the novel ISUP grading system is uncertain because studies investigating this correlation are lacking. Recently, a study on 44 PCa patients with a total of 101 PCa regions proposed that multiparametric MRI (mpMRI) radiomics in conjunction with a two-dimensional (2D) joint histogram generated from DCE images could potentially stratify PCa patients into low- (ISUP grade ≤ II) and high-ISUP grades (ISUP grade ≥ III) [14], implying that the use of DCE parameters alone may be sufficient for categorizing ISUP grades needing investigation.

Radical prostatectomy (RP) is a therapeutic procedure for clinically localized and locally advanced PCa that aims to surgically eradicate malignant tumors and ensure complete removal of the entire prostate gland and surrounding structures for optimal oncological control. However, positive surgical margins (PSMs) still occur after RP and may increase the risk of postoperative biochemical recurrence [15,16,17]. Since PSMs may suggest an adverse prognosis for PCa treatment, there is a need to identify risk factors for PSMs. Among studies that have investigated this topic [18,19,20,21,22], one factor identified was a GS greater than 6. A higher GS serves not only as a recognized risk factor for PSMs but also as a reliable indicator of increased aggressiveness in PCa. The complicated vascular microenvironment and functional characteristics of PCa can be assessed using DCE-MRI, which effectively reflects the GS and facilitates the evaluation of PCa aggressiveness. Hence, DCE-MRI parameters may exhibit significant associations with the occurrence of PSMs, thus offering potential insights into the prognosis of PCa. The integration of DCE-MRI parameters into clinical practice has the potential to enhance risk assessment and prognostic evaluation in the management of PCa.

This study aims to investigate the relationship between DCE-MRI parameters and the new ISUP grades as well as PSMs, as both ISUP grade and PSMs affect the prognosis of PCa after RP.

## 2. Materials and Methods

### 2.1. Patients

This retrospective study received approval from the Institutional Review Board (IRB), which waived the requirement for informed consent. From June 2016 to December 2017, 45 consecutive patients diagnosed with histologically proven PCa according to either prostate biopsy or transurethral resection of the prostate (TURP) prior to undergoing mpMRI of the prostate were enrolled in this study. All patients had detectable tumors demonstrated on prostate mpMRI and subsequently underwent RP within 90 days [23,24]. Notably, all of these patients had yet to receive any form of treatment for PCa before both mpMRI and RP other than TURP. Additionally, no concurrent malignancy was exhibited in the pathological specimens obtained from RP, ensuring that no other tumor interfered with the analysis of PCa.

### 2.2. MRI Technique and DCE Parameters on MRI

Images were acquired with a 3 Tesla (T) MRI scanner (Discovery MR750, GE Healthcare, Milwaukee, WI, USA). The imaging acquisition protocols consisted of various mpMRI sequences, including T2-weighted imaging (T2WI) in the three orthogonal planes (transverse, coronal, and sagittal), transverse T1-weighted imaging (T1WI), diffusion-weighted imaging (DWI), intravoxel incoherent motion (IVIM), and DCE imaging.

For T2WI, a fast spin echo (FSE) sequence was employed with the following parameters: repetition time (TR) ranging from 5800 to 6100 milliseconds (ms), echo time (TE) between 92 and 103 ms, slice thickness (ST) of 4 mm, matrix size of 384 × 320, and field of view (FOV) ranging from 180 × 180 to 240 × 240 mm^2^. The T1WI protocol employed the FSE sequence with the following parameters: TR 660 ms, TE 15 ms, ST 4 mm, matrix size 256 × 224, and FOV 180 × 180 mm^2^.

The IVIM protocol involved the acquisition of images using 8 different b values (0, 10, 30, 50, 80, 100, 400, 1000 s/mm^2^) and specific parameters, including a reduced FOV of 20 × 10 cm^2^, matrix size of 80 × 40, and TE of 53.4 ms. Following IVIM, DCE was conducted using a three-dimensional (3D) axial T1-weighted spoiled gradient-echo sequence. The DCE parameters included TR 2.6 ms, TE 1.1 ms, flip angle 13°, single excitations, matrix size 140 × 140, FOV 280 × 280 mm^2^, ST 4 mm, and a temporal resolution of 5.4 s for each phase. The DCE acquisition lasted for a total of 324 s (60 phases) with a standard dose (0.1 mmol/kg body weight) of gadopentetate dimeglumine (Gd-DTPA; Magnevist; Bayer-Schering, Burgess Hill, UK) injected at a rate of 3 mL/s.

A uroradiologist with over 20 years of experience meticulously reviewed all mpMRI images of each patient, especially the DCE images. The images that specifically demonstrated the dominant PCa tumor nodules were identified and selected from the DCE images of each patient. From these particular images, those exclusively showing the largest dominant tumor nodule were further selected. Subsequently, a region of interest (ROI) was manually delineated on the largest dominant nodule of PCa in each of these exclusive images with our in-house software constructed in MATLAB (R2015b; MathWorks, Inc., Natick, MA, USA). The ROI delineation process was carefully implemented to avoid areas presenting with liquefaction, necrosis, fibrosis, and calcification to the best of our ability. We then used our in-house software to spontaneously place these manually drawn ROIs in all exclusive DCE images onto the corresponding lesion sites in the DCE-derived parameter maps (i.e., *K^trans^*, *k_ep_*, and IAUC). Our in-house software is based on the well-established and widely accepted Tofts model, also known as the Kety model, for DCE-MRI analysis [25,26,27]. This model facilitates the computation of the transfer constant *K^trans^*, which describes the rate at which the contrast agent moves from blood plasma to the extracellular extravascular space (EES). It also computes the rate constant *k_ep_*, which indicates the velocity at which the contrast agent returns from the EES to the plasma, as well as the IAUC—an integrated parameter that considers both flow and permeability. Our software, having been rigorously tested and used in our institution for several years, has significantly augmented the findings of our prior studies [28,29].

**Figure 1:**
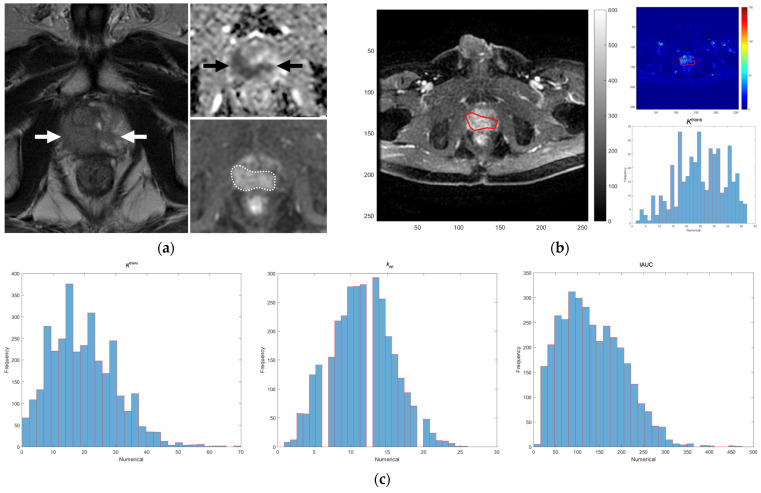
MRI images and histograms of the DCE parameters of a prostate cancer patient with ISUP grade V (GS = 5 + 4) on radical prostatectomy. (**a**) The dominant tumor involves both lobes of the prostate. It shows low signal intensity on the T2W image (white arrows), water restriction on the ADC map (black arrows), and early contrast enhancement on the DCE image (area in dotted line). (**b**) An ROI was delineated manually on the dominant tumor on the DCE image using our in-house software to automatically generate parametric maps and histograms of the *K^trans^*, *k_ep,_* and IAUC values simultaneously. (**c**) This process was repeated for other images of the dominant tumor in the MR image set to obtain the complete histograms of *K^trans^*, *k_ep_*, and IAUC of the dominant tumor for statistical analyses. ADC: apparent diffusion coefficient; DCE: dynamic contrast-enhanced; GS: Gleason score; ISUP: International Society of Urological Pathology; MRI: magnetic resonance imaging; ROI: region of interest.

Once these regions were established in the relevant parameter maps, histograms of the DCE parameters, that is, *K^trans^*, *k_ep_*, and IAUC, were automatically computed and generated within the delineated ROIs. By integrating all individual tumoral histograms derived from the corresponding parameter maps, our software obtained complete and comprehensive histograms of the entire largest dominant tumor within the prostate (Figure 1). The histogram values, i.e., minimum (min), median, and maximum (max), of *K^trans^*, *k_ep_*, and IAUC of the whole dominant tumor were recorded for statistical analyses.

### 2.3. ISUP Risk Groups and Surgical Margins on the Pathological Specimens

For every patient in our study, we documented pertinent clinical data at the point of diagnosis, including the patient’s age, titers of prostate-specific antigen (PSA), PSA density, and percentage of positive cores at prostate biopsy if carried out. Furthermore, we tried to discriminate the postoperative ISUP grades of PCas using the conclusive pathological findings obtained from RP specimens. The grading information was then used to stratify all patients into two different risk categories: a low-risk group, comprising ISUP grades I and II, and a high-risk group, consisting of ISUP grades III through V. Additionally, we recorded the surgical margin status on the RP specimens and classified it as either positive or negative in accordance with the definite results obtained from the comprehensive pathological examination. The location of the PSM was recorded using the method proposed in a previous study [30].

### 2.4. Statistical Analysis

Descriptive statistics for continuous variables are expressed as the median and interquartile range (IQR). Categorical variables are expressed as counts and proportions. The comparisons of age, PSA titer, PSA density, positive biopsy core percentage, and DCE parameters between the ISUP risk groups (high/low) and surgical margin statuses (positive/negative) were analyzed using the Mann-Whitney U test for continuous variables. Categorical variables were compared using the chi-square test or Fisher’s exact test. Univariate logistic regression analysis was used to analyze the association of age, PSA titer, PSA density, positive biopsy core percentage, and histogram values of DCE parameters with placement in the ISUP high-risk group and PSM positivity. Multivariate logistic regression using forward selection was used to further identify independent variables associated with the ISUP high-risk group and PSM. Statistical analyses were conducted utilizing SPSS Statistics version 25 (IBM, Armonk, NY, USA). The threshold for statistical significance was set at a two-tailed *p* value of less than 0.05.

## 3. Results

The distributions of age, PSA titers, PSA density, positive biopsy core percentages, DCE parameters, and pathological findings of PCa of the 45 patients are shown in Table 1. The PSA titers ranged from 2.01 ng/mL to 50.92 ng/mL at diagnosis, with 10 patients (22.22%) having a PSA titer less than 9 ng/mL and 2 patients (4.44%) having a PSA titer less than 4 ng/mL. Of the 22 pathological ISUP low-risk group patients, 4 were ISUP grade I, and 18 were grade II; of the 23 high-risk group patients, 19 were grade III, and 4 were grade V. Fifteen patients had PSMs on the pathological specimens, most commonly at the apex (7/15, 46.67%), followed by the posterior edge of the prostate (5/15, 33.33%).

There were no significant differences in age, PSA titers at diagnosis, PSA density, or positive biopsy core percentages between the two ISUP risk groups. Of the DCE parameters, there was a significant difference in *K^trans-max^, k_ep-median_*, and *k_ep-max_* between the two risk groups (*p* = 0.028, 0.019, and 0.033, respectively, Table 2). Univariate logistic regression analysis for all variables revealed that *K^trans-max^* and *k_ep-median_* were associated with the high-risk group (*p* =0.026 and 0.038, respectively, Table 3). After adjusting for the other variables, multivariate logistic regression analysis showed that a higher *K^trans-max^* was independently and significantly associated with the high-risk group (odds ratio [OR] = 1.032, 95% confidence interval [CI] = 1.005–1.060, *p* = 0.021).

PSMs were significantly more common (11/23, 47.83%) in the ISUP high-risk group than in the ISUP low-risk group (4/22, 18.18%, *p* = 0.035). There were significant differences in PSA titers, PSA density, positive biopsy core percentages, *K^trans-max^*, *k_ep-median_*, and *k*_ep-max_ between patients with and without PSMs (all *p* < 0.05, Table 2). Of these variables, univariate logistic regression analysis showed that PSA titers, positive biopsy core percentages, *K^trans-max^*, and *k*_ep-max_ were significantly associated with PSMs (all *p* < 0.05, Table 4). Further multivariate logistic regression analysis indicated that higher positive biopsy core percentages and *k*_ep-max_ were significant independent predictors of PSMs when controlling for other variables (OR = 1.035, 95% CI = 1.003–1.068, *p* = 0.032 and OR = 1.078, 95% CI = 1.012–1.148, *p* = 0.020, respectively).

## 4. Discussion

Angiogenesis may significantly influence the growth, metastasis, and prognosis of PCa. Evaluation of the angiogenic properties in PCa can yield prognostic markers to evaluate tumor aggressiveness, and DCE-MRI can noninvasively assess the hemodynamic status of the new vessels [6]. The most frequently used quantitative DCE-MRI parameters include *K^trans^*, the transfer rate constant of the contrast agent from intravascular to extravascular extracellular space (EES); v_e_, the EES volume fraction; and *k_ep_*, the rate constant for intravasation of the contrast agent from EES to intravascular space [27]. Angiogenesis may alter local blood flow, vascular permeability, and microvascular circulation in PCa, leading to increased *K^trans^* and *k_ep_* values in cancerous tissues [31]. V_e_ can serve as a marker of cellular density [32], where a low v_e_ reflects high cellular density.

The present study reveals that DCE parameters could reflect tumor aggressiveness when stratified into low-risk and high-risk ISUP groups. The quantitative DCE parameters *K^trans-max^* and *k_ep-median_* are associated with the pathological ISUP risk group. In particular, *K^trans-max^* is an independent significant predictor for the ISUP high-risk group (grades III–V). There have been several studies on the DCE parameters of PCa using 3T MRI that have supported the ability of *K^trans^* and *k_ep_* to classify tumor aggressiveness [7,8,9]. Their findings are in line with our results.

For instance, Vos et al. [7] assessed the quantitative parameters of DCE on 3T MRI for the aggressiveness of 41 PCas in the peripheral zones (PCa_PZs), categorized as low grade for Gleason grades 2 or 3 (n = 15), intermediate grade for secondary/tertiary Gleason grades 4 or 5 (n = 10), and high grade for primary Gleason grades 4 or 5 (n = 16). Among DCE quantitative parameters, *K^trans^* and *k_ep_* could potentially evaluate the risk of PCa_PZ, with *K^trans^*_75% and *k_ep_*_75% offering the highest discriminating performance between low-grade and intermediate-grade plus high-grade PCa_PZs (AUC of 0.72 for both) [7]. Cho et al. [8] evaluated DCE parameters on 3T MRI for 18 PCas in the PZ and 6 in the transitional zone (PCa_TZ). Their findings highlighted a statistically significant difference in the *K^trans^*_mean between tumors with a GS of 7 or less and those with a GS between 8 and 10 (both *p* < 0.05). This difference was also observed in the *k_ep_*_mean between the same GS groups (both *p* < 0.05). Furthermore, they reported a significant correlation between *K^trans^* and the GS (r = 0.623, *p* < 0.001), as well as between *k_ep_* and the GS (r = 0.562, *p* < 0.001).

In another study utilizing 3T MRI to assess DCE parameters in PCa, Baur et al. [9] found significant differences when comparing the median values of *K^trans^* and *k_ep_*. Specifically, they identified differences between high-grade PCa_PZs (ISUP grade ≧ 3) and a combined group of benign and nonhigh-grade PCa_PZs. The differences were significant, with *p* values of 0.005 and less than 0.001 for *K^trans^*_median and *k_ep_*_median, respectively. In brief, among DCE parameters, the histogram parameters of *K^trans^* and *k_ep_* can consistently discriminate the aggressiveness of PCa regardless of whether the old GS or new ISUP grading system is used. Recently, Urakami et al. [14] suggested that the integration of mpMRI radiomics with 2D histogram analysis derived from DCE-MRI might stratify patients with PCa into low- and high-risk groups based on their ISUP grades. All of these studies supported the use of DCE parameters as potential predictors for PCa risk stratification.

Our study demonstrated that PSMs are associated with an elevated PSA titer at diagnosis, higher positive biopsy core percentages, and increased values of *K^trans-max^* and *k*_ep-max_. However, after adjustment for other variables, multivariate logistic regression analysis indicated that higher positive biopsy core percentages and *k*_ep-max_ were independent and significant predictors for PSMs. In addition, we also found that PSMs were significantly more common in the high-risk group (ISUP grades III to V). In a recent study involving 179 patients who underwent preoperative 3T mpMRI to identify robust MRI-based risk predictors for PSMs in robotic-assisted RP, it was observed that patients with PSMs demonstrated significantly higher levels of PSA than those without PSMs [22]. This finding aligns with the results of our study, reinforcing the positive relationship between elevated PSA levels and the occurrence of PSM.

In addition to higher PSA titers, our study reaffirms the association between positive biopsy core percentages and the presence of PSMs. Previous studies have shown that positive biopsy core percentages are a significant predictor of PCa death and biochemical recurrence [33,34]. Moreover, in a recent study of 624 PCa patients undergoing nerve-sparing RP, van der Slot et al. [35] found that a positive biopsy core percentage was a significant predictor of a posterolateral PSM after RP. Yamashita et al. [36] found that a positive biopsy core percentage ≥ 60% was an independent risk factor for predicting pathological extraprostatic extension in patients who underwent robotic-assisted RP. Taking these findings together, positive biopsy core percentages may have potential in predicting surgical outcomes, underlining the importance of this clinical parameter in PCa prognosis.

The association of *K^trans-max^* and *k_ep-max_* with PSM was positive, especially that for *k_ep-max_*. The rationale behind this finding is straightforward and compelling from a clinical perspective. Increased values of *K^trans^* and *k_ep_* are observed in conjunction with high-risk PCa. In the present study, the high-risk group exhibited PSMs significantly more often. Thus, the higher *K^trans^* and *k_ep_* might also demonstrate a connection with an increased likelihood of PSMs. This finding indicates that these DCE-MRI parameters can stratify PCa risk and predict surgical outcomes. Further study of DCE parameters for PSM in PCa patients categorized by their PSA titers (e.g., >20 ng/mL, 10–20 ng/mL, and <10 ng/mL) may provide more information; however, more patients are required to obtain sufficient statistical power.

Studies have reported anatomic risk factors for PSMs, including tumor size and location, as well as prostate volume and apical depth, which can be revealed on MRI [18,19,20,21,22]. For example, the most common site of PSMs in this study was the apex, followed by the posterior edge, which is consistent with previous reports and determined by tumor location [21,22]. Nonetheless, the hypothesis of this study is that DCE parameters are risk factors for ISUP grades and PSMs on RP specimens, and the anatomic risk assessment on MRI is thus beyond the scope of this study and was not evaluated.

Recently, there has been an increasing trend toward the adoption of biparametric MRI (bpMRI), accompanied by a simultaneous decline in the utilization of DCE-MRI [37]. This transition can be attributed to the several advantages of bpMRI, such as eliminating adverse events, decreasing examination duration, and reducing costs [38]. From a diagnostic standpoint, bpMRI has comparable accuracy in detecting PCa to mpMRI [39]. However, a recent review revealed certain drawbacks in the studies advocating the substitution of mpMRI with bpMRI; many of these studies failed to provide transparent information about crucial DCE indications and did not conduct rigorous subanalyses focusing on image quality and reader experience [37]. This lack of comprehensive evaluation could bias the comparison between the two imaging modalities, casting doubt on the validity of the study conclusions.

In contrast, DCE-MRI continues to demonstrate its importance, especially in the management of PCa. For instance, in biologically targeted radiation therapy, DCE-MRI allows voxelwise tumor characterization, which contributes to the spatial understanding of tumor biology and aids in creating precise treatment plans [40]. Moreover, DCE-derived perfusion parameters are correlated more strongly with positron emission tomography standardized uptake values (PET SUV) than with ADC or T2WI, indicating their potential to inform biologically targeted radiation therapy planning [41]. Our study further demonstrated that DCE-MRI parameters can effectively distinguish between low- and high-risk cases based on ISUP grade. All these findings suggest the importance of DCE-MRI in treatment planning and risk stratification for PCa.

Similar to how the ISUP grade indicates the risk of PCa, the Prostate Imaging–Reporting and Data System Version 2 (PI-RADS v2) offers a five-point scale to denote the likelihood of clinically significant PCa (csPCa), which is defined by a GS ≥ 7, a tumor volume ≥ 0.5 cm^3^, or a tumor category ≥ T3 [42,43]. Studies have shown that as the PI-RADS v2 score increases, more severe histopathological findings become significantly more likely, including larger tumor volumes and a higher GS [44,45,46]. In a comprehensive analysis of 254 men with 323 PCa tumors, Afshari Mirak et al. [47] utilized whole-mount histopathology to evaluate the relationship between quantitative DCE-MRI parameters and PI-RADS v2 score. They found a significant positive correlation between certain DCE parameters (*K^trans^*, *k_ep_*, and IAUC) and the score; that is, higher DCE parameter values were related to higher PI-RADS v2 scores. In conclusion, the PI-RADS v2 score can stratify the risk of PCa, while DCE-MRI parameters have the potential to assess it.

While mpMRI is a favored diagnostic tool for PCa, recent findings have highlighted the comparable efficiency of other imaging modalities, notably ^68^Gallium-prostate-specific membrane antigen (^68^Ga-PSMA) positron emission tomography/computed tomography (PET/CT). A study involving 100 PCa patients evaluated the diagnostic accuracy of ^68^Ga-PSMA PET/CT-guided and mpMRI-guided targeted prostate biopsy (TPBx) for clinically significant PCa (csPCa: ISUP grade ≥ 2), in which 44 patients were classified as having csPCa [48].^68^ Ga-PSMA-TPBx had better diagnostic accuracy (84.9%) for csPCa than mpMRI-TPBx (76.9%). In another study, 80 patients with various ISUP grades (III–V) were diagnosed with csPCa (SUVmax cutoff ≥ 8 or PI-RADS v2 score ≥ 3) out of 125 high-risk PCa patients [49].^68^ Ga-PSMA PET/CT achieved a diagnostic accuracy of 92% for csPCa, surpassing the 86.2% of mpMRI. Moreover, ^68^Ga-PSMA PET/CT further identified metastases in 25% of csPCa patients. Similarly, a prior study reported ^68^Ga-PSMA PET/CT as a better choice in staging high-risk PCa because it might alter the therapeutic strategy [50]. Briefly, ^68^Ga-PSMA PET/CT and mpMRI are pivotal in diagnosing csPCa. However, the former seems particularly capable of staging PCa, and thus it may serve as a single modality for diagnosing and staging high-risk PCa.

There are limitations in the present work. First, the Tofts standard model assumes that the contrast agent is well mixed within the vasculature and the EES; thus, it is not valid for fibrotic and necrotic tissue [51]. During imaging interpretation, the experienced uroradiologist reviewed all mpMRI sequences and used T2-weighted imaging, diffusion-weighted imaging, and ADC mapping to exclude fibrotic and necrotic tissue. The ROI was manually delineated on the residual dominant tumor without fibrosis and necrosis on DCE-MRI. This process prevented the breakdown of the model assumption, and the results returned from the model might be correct. Second, this study adopted a retrospective design, focusing specifically on patients with PCa who underwent RP. There could be possible selection bias resulting from the recruitment of operable patients using the GS from biopsies/TURP as a reference for treatment selection. The final limitation in the present study is the relatively small number of included patients and lack of validation of the model. Consequently, a future prospective study enrolling a larger patient group whose data can be used for both model construction and validation should be conducted to provide a more robust foundation for clinical decision-making and further scientific inquiry.

## 5. Conclusions

In summary, we found that the quantitative parameters *K^trans-max^* and *k*_ep-max_, derived from DCE-MRI, are significantly associated with the ISUP grade and surgical margin status in pathological specimens on RP. *K^trans-max^* demonstrated good potential in predicting the final histological PCa risk group on RP specimens. Interestingly, in our study, the significant predictors for PSMs were a higher positive biopsy core percentage and *k*_ep-max_. The primary advantage of these quantitative DCE-MRI parameters is their ability to provide complementary information for the assessment of PCa aggressiveness and prognosis, thereby contributing to a complete and effective management plan for PCa and to the future of tailored and personalized treatment.

## Figures and Tables

**Table 1:** Clinical Characteristics, DCE Parameters, and Pathological Outcomes of the 45 Patients with Prostate Cancer.

Variables	
Clinical parameters	
Age (years)	66.000 (63.000–71.000)
PSA at diagnosis (ng/mL)	14.180 (9.075–20.895)
PSA density (ng/mL/mL)	0.414 (0.216–0.660)
Positive biopsy core (%)	33.333 (8.330–50.000)
DCE parameters	
*K^trans-min^* (min^−1^)	4.000 (2.000–7.000)
*K^trans-median^* (min^−1^)	19.000 (13.500–28.000)
*K^trans-max^* (min^−1^)	55.000 (34.500–78.000)
*k_ep-min_* (min^−1^)	2.000 (1.500–4.000)
*k_ep-median_* (min^−1^)	13.000 (9.250–14.000)
*k_ep-max_* (min^−1^)	25.000 (20.000–33.000)
IAUC*_-min_* (mM·s)	39.000 (19.500–60.500)
IAUC*_-median_* (mM·s)	137.000 (114.000–191.500)
IAUC*_-max_* (mM·s)	286.000 (210.500–411.000)
Outcomes from radical prostatectomy specimens	
ISUP risk group	
Low risk (I–II)	22 (48.889%)
High risk (III–V)	23 (51.111%)
Surgical margins	
Positive	15 (33.333%)
Negative	30 (66.667%)

Continuous variables are expressed as the median and interquartile range (IQR). Categorical variables are expressed as counts (%). DCE: dynamic contrast-enhanced; ISUP: International Society of Urological Pathology; max: maximum; min: minimum; mL: millimeter; mM: millimolar; ng: nanogram; PSA: prostate-specific antigen; s: second.

**Table 2:** Comparisons of Age, PSA Level at Diagnosis and DCE Parameters between the Two ISUP and Surgical Margin Groups.

Variables	ISUP Risk Group		*p*	Surgical Margin on the Pathological Specimens		*p*
	Low Risk (n = 22)	High Risk (n = 23)		Negative (n = 30)	Positive (n = 15)	
Clinical parameters						
Age (years)	65.500 (63.000–71.000)	66.000 (61.000–71.000)	0.838	66.500 (63.000–71.000)	66.000 (60.000–69.000)	0.405
PSA at diagnosis (ng/mL)	11.750 (8.260–17.390)	14.510 (9.120–21.370)	0.586	11.450 (8.260–15.540)	16.180 (10.120–32.710)	0.041 *
PSA density (ng/mL/mL)	0.405 (0.201–0.555)	0.471 (0.212–0.709)	0.570	0.306 (0.186–0.498)	0.562 (0.278–0.794)	0.043 *
Positive biopsy cores (%)	25.000 (8.330–54.165)	33.330 (10.415–50.000)	0.733	16.670 (8.330–33.330)	45.835 (27.080–62.498)	0.049 *
DCE parameters						
*K^trans-min^* (min^−1^)	5.000 (2.000–8.250)	3.000 (2.000–7.000)	0.490	5.000 (2.000–7.250)	3.000 (2.000–7.000)	0.379
*K^trans-median^* (min^−1^)	17.250 (13.000–26.000)	22.000 (15.000–28.000)	0.246	16.750 (13.000–27.000)	25.000 (17.000–28.000)	0.159
*K^trans-max^* (min^−1^)	43.000 (26.500–62.500)	70.000 (39.000–83.000)	0.028 *	42.000 (31.750–64.750)	79.000 (56.000–91.000)	0.010 *
*k_ep-min_* (min^−1^)	2.000 (1.000–4.000)	3.000 (2.000–5.000)	0.508	2.000 (1.000–4.250)	3.000 (2.000–4.000)	0.530
*k_ep-median_* (min^−1^)	11.000 (8.750–13.625)	14.000 (10.000–15.000)	0.019 *	11.000 (9.000–14.000)	14.000 (13.000–15.000)	0.013 *
*k_ep-max_* (min^−1^)	21.500 (16.500–29.750)	27.000 (22.000–37.000)	0.033 *	22.500 (20.000–27.500)	36.000 (21.000–44.000)	0.017 *
IAUC*_-min_* (mM·s)	43.500 (25.500–77.500)	32.000 (18.000–60.000)	0.364	40.500 (23.000–60.250)	32.000 (18.000–65.000)	0.485
IAUC*_-median_* (mM·s)	129.500 (96.000–183.000)	150.000 (115.000–234.000)	0.433	124.750 (102.500–183.000)	177.000 (128.000–234.000)	0.075
IAUC*_-max_* (mM·s)	247.000 (187.750–373.750)	150.000 (115.000–234.000)	0.059	259.500 (204.250–368.750)	366.000 (297.000–425.000)	0.060

Continuous variables are expressed as the median and interquartile range (IQR). DCE: dynamic contrast-enhanced; ISUP: International Society of Urological Pathology; max: maximum; min: minimum; mL: millimeter; mM: millimolar; ng: nanogram; PSA: prostate-specific antigen; s: second. *: statistically significant at a two-tailed *p* value of less than 0.05.

**Table 3:** Univariate and Multivariate Logistic Regression Analysis for Variables Associated with the ISUP High-Risk Group.

Variables	Univariate			*p*	Multivariate			*p*
	B	S.E.	OR (95% CI)		B	S.E.	OR (95% CI)	
Age (years)	−0.022	0.053	0.978 (0.881–1.087)	0.684				
PSA at diagnosis (ng/mL)	0.016	0.028	1.016 (0.963–1.073)	0.557				
PSA density (ng/mL/mL)	0.793	0.950	2.211 (0.344–14.227)	0.404				
Positive biopsy cores (%)	0.001	0.013	1.001 (0.976–1.027)	0.943				
DCE parameters								
*K^trans-min^* (min^−1^)	−0.022	0.055	0.978 (0.879–1.089)	0.686				
*K^trans-median^* (min^−1^)	0.039	0.035	1.040 (0.971–1.112)	0.263				
*K^trans-max^* (min^−1^)	0.029	0.013	1.030 (1.004–1.056)	0.026 *	0.031	0.014	1.032 (1.005–1.060)	0.021 *
*k_ep-min_* (min^−1^)	0.076	0.140	1.079 (0.820–1.419)	0.588				
*k_ep-median_* (min^−1^)	0.204	0.099	1.227 (1.011–1.488)	0.038 *				
*k_ep-max_* (min^−1^)	0.035	0.027	1.035 (0.982–1.091)	0.198				
IAUC*_-min_* (mM·s)	−0.007	0.008	0.993 (0.977–1.009)	0.396				
IAUC*_-median_* (mM·s)	0.004	0.005	1.004 (0.994–1.014)	0.419				
IAUC*_-max_* (mM·s)	0.005	0.003	1.005 (1.000–1.011)	0.060				

B: regression coefficient; CI: confidence interval; ISUP: International Society of Urological Pathology; max: maximum; min: minimum; mL: millimeter; mM: millimolar; ng: nanogram; OR: odds ratio; PSA: prostate-specific antigen; S.E.: standard error: s: second. *: statistically significant at a two-tailed *p* value of less than 0.05.

**Table 4:** Univariate and Multivariate Logistic Regression Analysis for Variables Associated with Positive Surgical Margin.

Variables	Univariate			*p*	Multivariate			*p*
	B	S.E.	OR (95% CI)		B	S.E.	OR (95% CI)	
Age (years)	−0.052	0.057	0.949 (0.848–1.062)	0.360				
PSA at diagnosis (ng/mL)	0.066	0.031	1.068 (1.004–1.135)	0.036 *				
PSA density (ng/mL/mL)	1.418	0.988	4.129 (0.596–28.606)	0.151				
Positive biopsy cores (%)	0.030	0.015	1.030 (1.001–1.061)	0.045 *	0.034	0.016	1.035 (1.003–1.068)	0.032 *
DCE parameters								
*K^trans-min^* (min^−1^)	−0.049	0.066	0.952 (0.837–1.083)	0.453				
*K^trans-median^* (min^−1^)	0.033	0.035	1.034 (0.965–1.108)	0.343				
*K^trans-max^* (min^−1^)	0.032	0.014	1.032 (1.005–1.060)	0.019 *				
*k_ep-min_* (min^−1^)	−0.007	0.146	0.993 (0.745–1.323)	0.961				
*k_ep-median_* (min^−1^)	0.033	0.035	1.034 (0.965–1.108)	0.343				
*k_ep-max_* (min^−1^)	0.074	0.031	1.076 (1.013–1.144)	0.018 *	0.075	0.032	1.078 (1.012–1.148)	0.020 *
IAUC*_-min_* (mM·s)	−0.007	0.009	0.993 (0.976–1.012)	0.475				
IAUC*_-median_* (mM·s)	0.008	0.005	1.008 (0.998–1.018)	0.125				
IAUC*_-max_* (mM·s)	0.005	0.003	1.005 (1.000–1.010)	0.065				

B: regression coefficient; CI: confidence interval; max: maximum; min: minimum; mL: millimeter; mM: millimolar; ng: nanogram; OR: odds ratio; PSA: prostate-specific antigen; S.E.: standard error; s: second. *: statistically significant at a two-tailed *p* value of less than 0.05.

## Data Availability

The data presented in this study can be obtained by contacting the corresponding author upon request.
